# Prioritization of Susceptibility Genes for Ectopic Pregnancy by Gene Network Analysis

**DOI:** 10.3390/ijms17020191

**Published:** 2016-02-01

**Authors:** Ji-Long Liu, Miao Zhao

**Affiliations:** College of Veterinary Medicine, South China Agricultural University, Guangzhou 510642, China; zm1586186423@126.com

**Keywords:** ectopic pregnancy, pathogenesis, text mining, gene prioritization

## Abstract

Ectopic pregnancy is a very dangerous complication of pregnancy, affecting 1%–2% of all reported pregnancies. Due to ethical constraints on human biopsies and the lack of suitable animal models, there has been little success in identifying functionally important genes in the pathogenesis of ectopic pregnancy. In the present study, we developed a random walk–based computational method named TM-rank to prioritize ectopic pregnancy–related genes based on text mining data and gene network information. Using a defined threshold value, we identified five top-ranked genes: *VEGFA* (vascular endothelial growth factor A), *IL8* (interleukin 8), *IL6* (interleukin 6), *ESR1* (estrogen receptor 1) and *EGFR* (epidermal growth factor receptor). These genes are promising candidate genes that can serve as useful diagnostic biomarkers and therapeutic targets. Our approach represents a novel strategy for prioritizing disease susceptibility genes.

## 1. Introduction

Ectopic pregnancy is defined as embryo implantation outside of the uterine cavity, with the most common site being the Fallopian tube [1]. Its incidence is 1%–2% of all reported pregnancies [2]. Ectopic pregnancy is a very dangerous complication of pregnancy, which accounts for 10% of all maternal deaths during the early stage of pregnancy [2]. Notably, unlike humans, the major type of ectopic pregnancy in animals is abdominal pregnancy, and tubal pregnancy is rather rare [3]. The pathogenesis of ectopic pregnancy in humans is not fully understood.

It has been suggested that both impaired embryo transport in the Fallopian tube and abnormal tubal environment may contribute to the pathogenesis of ectopic pregnancy. The major risk factors for ectopic pregnancy include tubal infection and smoking [4,5]. These two conditions initiate inflammation within the Fallopian tube, leading to embryo retention by disrupting smooth muscle contractility and ciliary beat frequency [6]. The inflammatory environment may also provide necessary pro-implantation factors with roles in the establishment of Fallopian tube receptivity for the retained embryo [7]. A number of genes have been implicated in ectopic pregnancy [8,9,10]. However, due to ethical constraints on human biopsies and the lack of suitable animal models, most studies have focused on altered tubal gene or protein expression by comparing ectopic pregnancy with normal controls; thus, these studies are largely descriptive and speculative. There has been little success in identifying functionally important genes in the pathogenesis of ectopic pregnancy.

Recently, text mining methodology has been implemented [11], making it possible to retrieve all ectopic pregnancy candidate genes from published experimental results in an automated way. Because functional validation of these candidates using experimental methods is not feasible for now, computational methods may provide a useful option for prioritization of the most likely susceptibility genes. Among all computational methods, the random walk algorithm, which incorporates topological properties of the gene network, has attracted more and more attention in recent years [12,13]. It is a variant of the PageRank algorithm used by the Google search engine to rank search results on the world-wide web [14]. In the present study, we developed a random walk–based strategy named TM-rank to prioritize ectopic pregnancy–related genes by combining text mining data and gene network information. Our approach represents a novel strategy for prioritization of disease susceptibility genes.

## 2. Results

### 2.1. Identification of Susceptibility Genes for Ectopic Pregnancy by Using Text Mining

**Figure 1:**
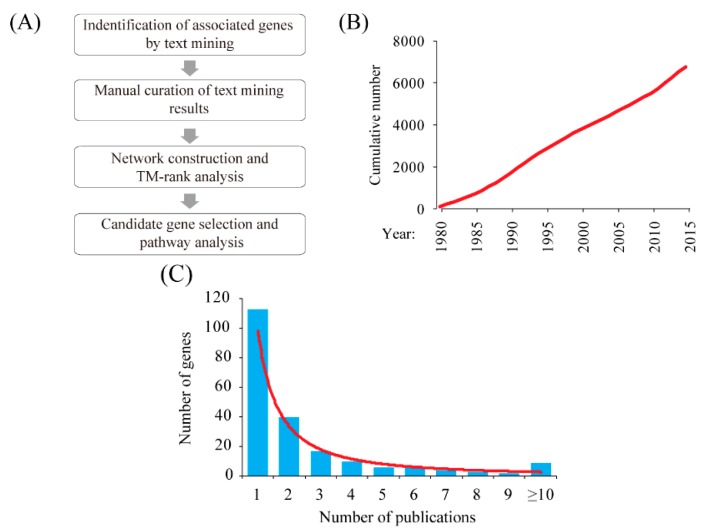
Systematic identification of susceptibility genes for ectopic pregnancy. (**A**) Overview of the experimental design; (**B**) Cumulative number of publications related to ectopic pregnancy by year (from 1980 to 2015); (**C**) Distribution of the number of publications per gene.

We carried out a key word search in the PubMed database and obtained 7363 publications related to ectopic pregnancy as a result (from January 1980 to October 2015). The titles and abstracts of these publications were fetched and analyzed through a computational pipeline (Figure 1A). Statistical analysis shows that the number of publications on ectopic pregnancy has been growing linearly in recent years (Figure 1B). From these publications, a total of 264 ectopic pregnancy–related genes were extracted (Figure 1C; Table S1).

### 2.2. Construction of Gene Network

**Figure 2:**
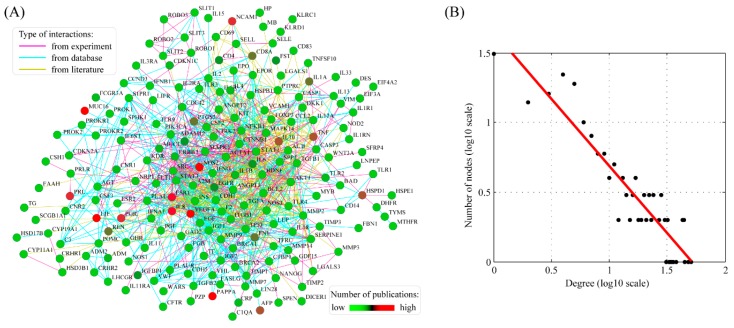
The gene network underlying ectopic pregnancy susceptibility genes. (**A**) The structure of the gene network generated by the STRING software. The number of publications for each gene was represented in color gradient with a scale bar; (**B**) Degree distribution of the gene network. The degree distribution follows a power law distribution. With 95% confidence interval, the scaling exponent is estimated to be 0.9614 ± 0.1346.

The gene network was generated by using the STRING (Search Tool for the Retrieval of INteracting Genes/proteins) database. With the combined score being set to 0.9, we obtained a network consisting of 198 nodes connected via 1007 edges (Figure 2A). Topological analysis showed that the network follows a power-law distribution (Figure 2B). In this network, we identified nine hub genes: *IL6* (interleukin 6), *SRC* (v-src sarcoma viral oncogene homolog), *STAT3* (signal transducer and activator of transcription 3), *TP53* (tumor protein p53), *VEGFA* (vascular endothelial growth factor A), *AKT1* (v-akt murine thymoma viral oncogene homolog 1), *PIK3CA* (phosphoinositide-3-kinase catalytic α polypeptide), *NFKB1* (nuclear factor of kappa light polypeptide gene enhancer in B-cells 1), *IGF1* (insulin-like growth factor 1), *CTNNB1* (catenin β 1), *TGFB1* (transforming growth factor β 1), *EGFR* (epidermal growth factor receptor), *INS* (insulin) and *MMP9* (matrix metallopeptidase 9).

### 2.3. Gene Prioritization by TM-Rank

**Figure 3:**
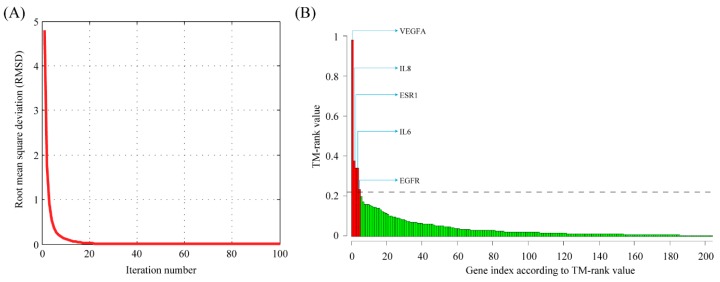
Prioritization of susceptibility genes for ectopic pregnancy by the TM-rank algorithm. (**A**) Convergence of the TM-rank algorithm. Convergence was confirmed by observing an exponential decrease in the RMSD (root mean square deviation) values during the sequential iteration; (**B**) Gene ranking by the TM-rank algorithm. Genes were sorted according to rank values in descending order. Top-ranked genes, which exceeded the mean plus two standard deviations, were labeled.

Initially, the gene rank (the importance of a gene to ectopic pregnancy) was determined by the number of publications for each gene. Considering the network underlying all ectopic pregnancy–related genes, we would like a gene to be highly ranked if it is linked to other highly ranked genes. Therefore, we developed the TM-rank algorithm, which extends the PageRank algorithm [14], for analyzing our text mining results. We demonstrated that the importance of a gene as determined by the number of publications could propagate fast through the entire network by iterative random walk (Figure 3A). Finally, genes were ranked according to TM-rank values in descending order. As a result, the top five genes exceeded a defined TM-rank cut-off (mean + 2 × SD = 0.1919). These five genes were: VEGFA (vascular endothelial growth factor A), IL8 (interleukin 8), IL6 (interleukin 6), ESR1 (estrogen receptor 1) and EGFR (epidermal growth factor receptor) (Figure 3B).

**Figure 4:**
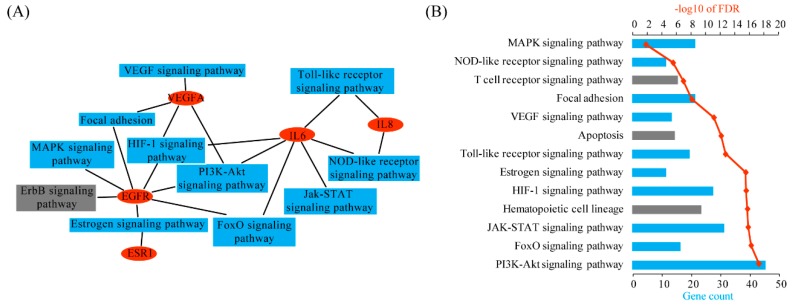
Pathway analysis. (**A**) Pathway assignment for the top five genes recommended by the TM-rank algorithm. This graph was generated by using the Cytoscape software. Ellipse nodes in red represent genes and rectangle nodes in blue represent pathways; (**B**) Pathway enrichment analysis for all the 264 genes identified by text mining. This analysis was performed by using the DAVID tool. The significance cutoff for FDR was set at 0.01.

By searching the KEGG (Kyoto Encyclopedia of Genes and Genomes) pathway database [15], these five genes are linked to 11 pathways (Figure 4A). EGFR is involved in seven pathways, namely the estrogen signaling pathway, focal adhesion pathway, ErbB signaling pathway, MAPK signaling pathway, PI3K-Akt signaling pathway, FoxO signaling pathway and HIF-1 signaling pathway. IL6 is associated with six pathways: the Jak-STAT signaling pathway, PI3K-Akt signaling pathway, HIF-1 signaling pathway, FoxO signaling pathway, NOD-like receptor signaling pathway and Toll-like receptor signaling pathway. Beyond the VEGF signaling pathway, VEGFA also plays a role in the PI3K-Akt signaling pathway, HIF-1 signaling pathway and focal adhesion pathway. IL8 participates in the NOD-like receptor signaling pathway and Toll-like receptor signaling pathway. ESR1 is the key component of the estrogen signaling pathway.

In addition, we performed pathway enrichment analysis on the complete list of 264 susceptibility genes by using the DAVID (Database for Annotation, Visualization and Integrated Discovery) tools. As a result, a total of 13 pathways, namely the PI3K-Akt signaling pathway, FoxO signaling pathway, Jak-STAT signaling pathway, hematopoietic cell lineage, HIF-1 signaling pathway, estrogen signaling pathway, Toll-like receptor signaling pathway, apoptosis, VEGF signaling pathway, focal adhesion, T cell receptor signaling pathway, NOD-like receptor signaling pathway, and MAPK signaling pathway, are significantly enriched (false discovery rate FDR < 0.05) (Figure 4B). Interestingly, all 11 pathways linked to top-ranked genes are overlapped with these 13 pathways enriched among all genes identified by text mining, indicating that our TM-rank algorithm is able to prioritize functionally important genes for ectopic pregnancy.

## 3. Discussion

Ectopic pregnancy is a very dangerous complication of pregnancy, the pathogenesis of which is not fully understood. There is a wealth of information that is hidden within published experimental results. In the present study we performed a text mining analysis of ectopic pregnancy–associated genes. A total of 264 genes were indentified from 7363 publications. Since a large number of publications were analyzed, our result may provide reasonably good coverage of the entire set of ectopic pregnancy–associated genes.

Interpretation of the text mining results is usually based on the number of publications for each gene. That is, the more publications a gene is cited in, the more important the gene is to ectopic pregnancy. The disadvantage is that genes are treated as separated entities and the interactions between genes are ignored. It has been shown that gene products or proteins usually form protein complexes to execute their functions instead of acting alone [16]. Hence, a gene is indicative of functional importance for its interacting partners, opening the possibility to integrate interaction information for gene prioritization. In the present study, a genome-wide gene network was constructed by using up-to-date interaction data available in the STRING database [17]. Using a threshold value of 0.9 for the combined score, we obtained a gene network consisting of 198 nodes connected via 1007 edges. So far, several studies have been conducted to incorporate the topology of the gene network in prioritization of disease candidate genes [18,19,20]. The main concern for these studies is that the incompleteness and noisiness of interaction data may affect the accuracy of the prioritization results. By weighting and integrating interaction data from high-throughput experiments and from the mining of databases and literature, the STRING database provides a comprehensive gene network at a reasonable false positive rate [17]. We expected that the use of the STRING database may alleviate this problem to a certain extent.

Using text mining data and gene network information as input, we developed a random walk–based algorithm named TM-rank to prioritize ectopic pregnancy–related genes. This algorithm is a variant of the PageRank algorithm used by the Google search engine to rank search results on the world-wide web [14]. Compared to previously described algorithms, our method is unique in that: (1) both text mining data and gene interaction information are used; (2) no training set of functionally confirmed genes is needed; and (3) valuable insights of genes with high susceptibility to ectopic pregnancy are recommended.

An arbitrary cutoff value for TM-rank is needed to select top-ranked genes. If an arbitrary cutoff of 0.1 is used, the number of selected genes will be 23. On the other hand, if the top 10 genes are selected, the TM-rank cutoff will be 0.15. In the present study, we used mean + 2 × SD, which is 0.1919, as the cutoff value. However, because the distribution of TM-rank is not a normal distribution (Kolmogorov-Smirnov goodness-of-fit test, *p* = 3.3 × 10^−27^), this cutoff is actually no better than any other one. Using this cutoff value, we identified a total of five top-ranked genes: *VEGFA* (vascular endothelial growth factor A, count = 21, degree = 44, TM-rank = 0.98909), *IL8* (interleukin 8, count = 9, degree = 31, TM-rank = 0.38012), *IL6* (interleukin 6, count = 5, degree = 51, TM-rank = 0.33978), *ESR1* (estrogen receptor 1, count = 9, degree = 24, TM-rank = 0.33905) and *EGFR* (epidermal growth factor receptor, count = 5, degree = 33, TM-rank = 0.23342). In contrast, the top five genes ranked by count of publications are (six genes actually, count ≥ 10): *MUC16* (mucin 16, count = 22, degree = 1, TM-rank = 0.13124), *VEGFA* (count = 21, degree = 44, TM-rank = 0.98909), *PSG1* (pregnancy specific β1-glycoprotein 1, count = 13, degree = 0, TM-rank = 0.031113), *NOS2* (nitric oxide synthase 2, count = 10, degree = 6, TM-rank = 0.083989), *LIF* (leukemia inhibitory factor, count = 10, degree = 4, TM-rank = 0.059718), and *PAPPA* (pregnancy-associated plasma protein A, count = 10, degree = 2, TM-rank = 0.034855). Of these genes, only *VEGF* is among the top five genes recommended by TM-rank. Therefore, our TM-rank algorithm provides a balance between the count of publication and the degree in the network.

These top five genes recommended by TM-rank can serve as useful diagnostic biomarkers and therapeutic targets. Serum VEGF is reported to be higher in ectopic pregnancy compared with normal pregnancy, indicating that VEGF may be a useful diagnostic biomarker for ectopic pregnancy [21]. Moreover, VEGF has been shown to be up-regulated at the implantation site compared to the non-implantation site of the Fallopian tube in ectopic pregnancy [22]. It is thought that elevated expression of VEGF may contribute to ectopic pregnancy by enhancing the supply of oxygen to the embryo via angiogenesis [23]. IL-6 and IL-8 are inflammatory cytokines and both of them are highly induced in the Fallopian tube upon chlamydial infection [24] as well as ectopic implantation [25]. Embryo implantation into the uterus is known to induce a local inflammatory response in a similar way as leukocytes accumulate at inflammatory sites [26]. It is likely that IL-6 and IL-8, induced by inflammation in the Fallopian tube, may provide signals to the embryo and promote tubal implantation. As the predominant estrogen receptor subtype in the Fallopian tube [27], ESR1 has been shown to serve as a crucial regulator in Fallopian tube development [28]. Exogenous estrogen treatment enhances embryo transport in the mouse Fallopian tube [29,30]. Interestingly, ESR1 protein has been shown to be absent in the Fallopian tube in human ectopic pregnancy [31], suggesting a role of ESR1 in the pathogenesis of ectopic pregnancy. EGFR is highly expressed at the ectopic implantation site [32]. It has been demonstrated that EGFR is a promising target for the treatment of ectopic pregnancy [33].

Unlike the supervised method with a positive training set, our un-supervised prioritization method is very difficult to provide validation data for. Wet experiments are often used; however, due to ethical constraints on human biopsies and the lack of suitable animal models, experimental validation is not a feasible option in this case. Alternatively, we performed pathway enrichment analysis on the complete list of 264 susceptibility genes by using the DAVID tools. A total of 13 enriched pathways were identified. In contrast, the top five genes selected by TM-rank are linked to 11 pathways. Interestingly, all 11 pathways are overlapped with the 13 enriched pathways, except for the ErbB signaling pathway. This result might provide validity of our methodology.

A limitation for text mining–based strategies is that there is no chance to discover new genes. In order to solve this problem, we enlarged the network by including new genes that are not reported to be involved in ectopic pregnancy. According to the rule of “guilty-by-association”, these new genes may be potential susceptibility genes. Finally, we made a list of 10 new genes, all of which have more than two connections with known genes (Table S2).

In conclusion, we developed a random walk–based algorithm named TM-rank to prioritize ectopic pregnancy–related genes based on text mining data and gene network information. Application of the TM-rank methodology yielded promising candidate genes that can serve as useful diagnostic biomarkers and therapeutic targets. Our study on ectopic pregnancy can act as an illustrative example for broader use in various diseases.

## 4. Materials and Methods

### 4.1. Text Mining

The PubMed database [34] was used as a source of publications for text mining. We conducted a search with the following combinations of query key words: “ectopic pregnancy” OR “tubal pregnancy” OR “eccyesis”. The search tag “(Title/Abstract)” was added after each key word. In order to make information extraction more precise, the relevant publications were retrieved in XML format with content enclosed within XML tag pairs. For each article, titles and abstract texts were fetched and transformed into the PubTator format [35] using in-house PERL scripts.

Text mining was performed by using the GNormPlus pipeline [36]. GNormPlus includes two modules: gene mention recognition and gene name normalization, respectively. Gene mentions were detected by using the CRF++ library [37]. Gene name normalization was performed by GenNorm [38]. The GNormPlus pipeline has achieved a precision of 87.1% and a recall of 86.4% [36]. To ensure accuracy, we manually checked each entry from the output of GNormPlus. A total of 1864 false positive errors out of a total of 6771 entries were identified. Thus, the observed precision was 4907/6771 = 72.47%. Finally, all the curated entries were summarized and a full gene list associated with ectopic pregnancy was compiled.

### 4.2. Construction of Gene Network

The STRING database v10.0 [17] was used to create gene network. The minimum combined score was set to 0.900 (highest confidence). The Cytoscape software [39] was applied for visualization and analysis of the gene network. The degree for each gene was analyzed by NetworkAnalyzer [40]. The threshold value for hub genes was defined as the mean plus two standard deviations.

### 4.3. The TM-Rank Algorithm

In order to prioritize susceptibility genes for ectopic pregnancy, TM-rank adopted a random walk in the gene network to infer which genes are most important. We followed a general implementation of a random walk in an iterative form:

(1) $$r^{t+1}=(1-d)Wr^{t}+dr^{0}$$

where *r^t^*^+1^ and *r^t^* are the TM-rank vectors in which the *i*-th element represents the TM-rank for gene *i* at time steps *t* and *t*+1, respectively. The parameter *d* is the restart probability which is set to 0.15 in this study. The vector *r*° is the initial vector. Taking the number of publications into consideration, we biased the initial rank vector to the normalized number of publications for each gene. Assuming the total number of genes in the gene network is *n*, then the column-normalized network adjacency matrix can be written as:

(2) $$W=\left[\begin{array}{ccc} w_{11} & \cdots & w_{1n}\\ \cdots & \ddots & \cdots \\ w_{n1} & \cdots & w_{nn} \end{array}\right]$$

For a given gene *i*, let *N_i_* be the node set of immediate interacting neighbors of node *i* and *c_i_* be the number of publications for gene *i*. Then *w_ij_* is defined as:

(3) $$w_{ij}=\{\begin{array}{c} \frac{c_{j}}{\sum_{k\in N_{i}}c_{k}}\;,\text{ if gene }i\text{ interacts with gene }j\\ 0,\text{ otherwise} \end{array}$$

The TM-rank algorithm was implemented in MATLAB language (MathWorks, MA, USA).

## Supplementary Materials

Supplementary materials can be found at http://www.mdpi.com/1422-0067/17/2/191/s1.
